# Fast access and early ligation of the renal pedicle significantly facilitates retroperitoneal laparoscopic radical nephrectomy procedures: modified laparoscopic radical nephrectomy

**DOI:** 10.1186/1477-7819-11-27

**Published:** 2013-01-30

**Authors:** Qing Yang, Jun Du, Zhi-Hua Zhao, Xu-Sheng Chen, Lei Zhou, Xin Yao

**Affiliations:** 1 Department of Genitourinary Oncology, Tianjin Medical University Cancer Institute and Hospital, Key Laboratory of Cancer Prevention and Therapy, Huanhuxi Road, Hexi District, Tianjin, 300060, People’s Republic of China

**Keywords:** Retroperitoneal laparoscopic nephrectomy, Technical modification, Operation time, Experience, Surgical outcomes

## Abstract

**Background:**

The objective of this study was to develop a modified retroperitoneal laparoscopic nephrectomy and compare its results with the previous technique.

**Methods:**

One hundred retroperitoneal laparoscopic nephrectomies were performed from February 2007 to October 2011. The previous technique was performed in 60 cases (Group 1). The modified technique (*n* = 40) included fast access to the renal pedicle according to several anatomic landmarks and early ligation of renal vessels (Group 2). The mean operation time, mean blood loss, duration of hospital stay conversion rate and complication rate were compared between the groups.

**Results:**

No significant differences were detected regarding mean patient age, mean body mass index, and tumor size between the two groups (*P* >0.05). The mean operation time was 59.5 ± 20.0 and 39.5 ± 17.5 minutes, respectively, in Groups 1 and 2 (*P* <0.001). The mean intraoperative blood loss was 147 ± 35 and 100 ± 25 ml, respectively, in Groups 1 and 2 (*P* <0.001). No significant differences were detected regarding the conversion rate and the complication rate between the two groups (*P* >0.05).

**Conclusions:**

Early ligature using fast access to the renal vessels during retroperitoneal laparoscopic radical nephrectomy contributed to less operation time and intraoperative blood loss compared with the previous technique. In addition, the modified technique permits the procedure to be performed following the principles of open radical nephrectomy.

## Background

Laparoscopic radical nephrectomy has been accepted as the gold standard for the treatment of renal cell carcinoma confined to the kidney without deteriorating the oncologic outcome [1-8]. Traditional open radical nephrectomy as described by Robson explains various stages; one of the most fundamental steps is the early ligature of the renal artery to prevent diffusion of cancer cells [6,9,10]. In addition, early ligation of renal vessels could facilitate the dissection of the kidney in further steps due to less bleeding and dissociation of the kidney from the renal pedicle. The principal goal of laparoscopy is to reproduce the principle of open surgery whilst achieving a minimally invasive treatment [1,2,11]. However, owing to the difficulty of direct access to renal vessels, regardless of whether the transperitoneal, retroperitoneal or hand-assisted approach is employed, this principle is often not performed in laparoscopic radical nephrectomy [3,7,12-14]. Several authors recently tried to directly access the renal artery using transperitoneal laparoscopic radical nephrectomy [15-18]. Porpiglia and colleagues developed a modified technique of direct access to and early ligation of the renal artery at the level of the Treitz ligament, permitting the surgeon to follow the classic steps and principles of radical nephrectomy, which have driven open surgery techniques for several years [15,17]. Unfortunately, fast access and early ligation of renal vessels in retroperitoneal laparoscopic radical nephrectomy have not been reported.

In the present study, we describe fast access and early ligation of the renal pedicle during retroperitoneal laparoscopic radical nephrectomy and we compare this technique with the standard one.

## Materials and methods

### Subjects

We performed 100 retroperitoneal laparoscopic radical nephrectomies at our institution between February 2007 and October 2011. Ultrasonography and computed tomography were used to identify the renal mass and characterize the surrounding anatomy. The same experienced laparoscopic team executed all procedures. Patients in whom lymph node metastasis and renal vein thrombus were diagnosed were not included for laparoscopic intervention. All tumors were clinically diagnosed as stage T1. Tumor size ranged from 25 to 66 mm (mean 43 mm). All tumors were not suitable for partial nephrectomy. In detail, 78 patients presenting Preoperative Aspects and Dimensions Used for an Anatomical score ≥8 points, 21 patients refusing to receive partial nephrectomy and one patient with myelodysplastic syndrome were not suitable for partial nephrectomy. Eleven cases had a history of intra-abdominal surgery, but none of them had a history of retroperitoneal surgery. In 47 patients the tumor was on the left side, and in 52 patients it was on the right side.

### Operation preparation and trocar placement

Preoperative mechanical bowel preparation was performed. The operation was performed by adopting tracheal intubation for general anesthesia. The patients were maintained in the lateral decubitus position. Patients’ affected sides were in a semi-oblique position and at 90° with the bed. A 1.5 cm transverse incision was made 2 cm above the crista iliaca in the midaxillary. Skin and subcutaneous tissue were cut in sequence. The muscle and lumbodorsal fasciae were then dissected with vessel forceps, and the retroperitoneal space was entered by blunt finger dissection. A 12 mm trocar was placed in this site (site A). A suture was placed in this incision to avoid air entering the retroperitoneal space. Under direct vision, a 12 mm trocar was placed in site B (below the costa margin in the posterior axillary line) and a 5 mm trocar was placed in site C (below the costa margin in the anterior axillary line).

### Previous surgical technique

The first 60 cases were performed using previous retroperitoneal radical nephrectomy as described previously [13,14]. The surgical steps included: the dorsal side and lateral side of the kidney being dissociated adequately, then the abdominal side and medial side, and finally the lower and upper pole; identification and dissection of the renal hilum; identification and skeletonization of the renal artery and vein; occlusion and division of these vessels by endoclips (Hem-o-Lok polymer clip; Weck Closure Systems, Research Triangle Park, NC, USA); dissection of the ureter; completion of nephrectomy dissection; and finally entrapment of the kidney in the endobag and removal of the specimen. A drain was left in place.

### Modified surgical technique

**Figure 1:**
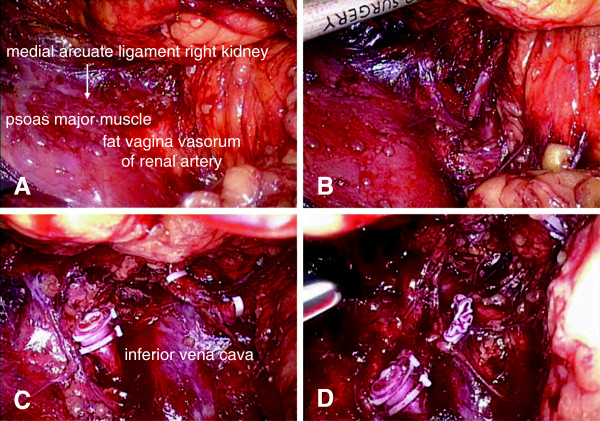
**Demonstration of modified technique. (A)** A demi-mounded apophysis at the renal pedicle near the psoas major muscle; this apophysis is the fat vagina vasorum of the renal artery. **(B), (C)** Renal artery can be exposed by dissecting and cutting fat and fibrous vagina vasorum of renal artery. **(D)** Renal vein is dissected and cut.

As our experience increased, in the following 40 cases we modified our technique. Patient positioning and trocar sites were the same as explained above. To identify the renal pedicle, we noticed that it was not necessary to mobilize the abdominal side and medial side, upper and lower pole of the kidney. After mobilizing the dorsal side and lateral side of the kidney adequately, we could recognize the location of the renal pedicle definitively according to several anatomic landmarks. In brief, after adequate mobilization of the back side and lateral side, the kidney was pushed directly to the abdominal side, while the position of the renal pedicle could not be moved due to the pulling of renal vessels. Meanwhile, we could observe the eminence of the renal pedicle near the inner side of medial arcuate ligament, and this eminence was the fat and fibrous vagina vasorum of the renal artery – renal vessels could be exposed by dissecting and cutting these tissues. The renal vessels were then ligated, and cut as explained above. The abdominal side, lower and upper pole, and medial side of the kidney were then extensively mobilized. The following surgical steps for ureter dissection, completion of nephrectomy dissection and kidney entrapment were the same as described above (Figure 1).

Group 1 (previous technique) included 60 cases and Group 2 (modified technique) included 40 cases. The mean operation time, mean blood loss, and duration of hospital stay were compared between the groups. All procedures were performed by a single surgeon (YQ) starting from trocar insertion and placement of the kidney into the endobag. Because extraction of the specimens and closure of the incisions and port sites were performed by the assistant (a urologist or a urology resident) in most of the procedures, the operation time was calculated between trocar insertion and placement of the kidney into the endobag, which was performed in all cases by a single surgeon (YQ). The tumor size was measured postoperatively during pathologic evaluation. Statistical analysis was performed using Student *t* tests and Mann–Whitney U tests. *P* <0.05 was regarded as statistically significant.

## Results

The preoperative data of Groups 1 and 2 are summarized in Table 1. No significant differences were found among them. Mean intraoperative blood loss was 147 ± 35 and 100 ± 25 ml, respectively, in Groups 1 and 2 (*P* <0.001). The duration of hospital stay was 5.2 ± 1.2 and 4.6 ± 1.0 days, respectively, in Groups 1 and 2 (*P* = 0.015). The mean operation time was 59.5 ± 20.0 and 39.5 ± 17.5 minutes, respectively, in Groups 1 and 2 (*P* <0.001) (Table 2).

**Table 1:** Preoperative data for patients who underwent retroperitoneal laparoscopic nephrectomies using the standard and modified techniques

	**Previous technique (Group 1)**	**Modified technique (Group 2)**	***P*value**
Sex			0.734
Male	38	26	
Female	26	14	
Mean age (years)	51.5 ± 4.6	53.0 ± 3.6	0.097
Mean body mass index (kg/m^2^)	25.5 ± 4.9	25.0 ± 4.8	0.633
Tumor location			0.550
Left	34	23	
Right	26	17	
Tumor size (cm)	5.0 ± 0.8	4.8 ± 0.8	0.256

Data presented as *n* or mean ± standard deviation.

**Table 2:** Perioperative data for patients who underwent retroperitoneal laparoscopic nephrectomies using the standard and modified techniques

**Variable**	**Previous technique (Group 1)**	**Modified technique (Group 2)**	***P *value**
Total cases (*n*)	60	40	
Operative time (minutes)	59.5 ± 20.0	39.5 ± 17.5	<0.001
Evaluated blood loss (ml)	147 ± 35	100 ± 25	<0.001
Hospital stay (days)	5.2 ± 1.2	4.6 ± 1.0	0.015
Conversion rate (%)	0	0	1.000
Complication rate (%)	3.3 (2/60)	2.5 (1/40)	0.649

Data presented as mean ± standard deviation unless stated otherwise.

All procedures were completed successfully. No procedure required conversion to open surgery. Renal vein injury occurred in two patients of Group 1 and in one patient of Group 2 during renal pedicle dissection. Bleeding due to renal vein injury was controlled laparoscopically in all three patients.

A description of our modified technique is shown in Figure 1.

## Discussion

Laparoscopy has become diffused in the treatment of many urological diseases; the most effective use of laparoscopy has been experienced in radical nephrectomy for tumors confined to the kidney and is the preferred operative approach for most urologists [1-5,11]. Dunn and colleagues reported that laparoscopic surgery could efficiently lower the intraoperative blood loss, postoperative analgesic requirement and hospital stay [19]. Although these techniques are widely used and have been the subject of many variations, there is still a wide margin for further development. Many surgeons are now focusing on modification of the laparoscopic surgical technique.

In our study, Group 1 involved the previous technique. After entirely mobilization of the kidney (dorsal and abdominal side, upper and lower pole), renal vessels were dissected, ligated and divided. However, in our modified technique, after mobilizing the dorsal side and lateral side of the kidney adequately, we could recognize the location of the renal pedicle definitely according to several anatomic landmarks. The renal vessels were then manipulated as explained above. According to our experience, the critical points of fast access to the renal pedicle can be summarized as follows: extensively mobilize the lateral and dorsal side of kidney to the inner side of the psoas major muscle; after full mobilization of the kidney’s lateral and dorsal side, the eminence of the renal pedicle is usually located near the inner side of the medial arcuate ligament; the position of the renal pedicle could not be moved due to the pulling of renal vessels; and the eminence was actually the fat and fibrous vagina vasorum of renal artery. In brief, the important anatomic landmarks during this process included the psoas major muscle, the medial arcuate ligament and the eminence of the renal pedicle.

To reproduce the principles of open radical nephrectomy and to achieve early ligature for the treatment of renal cell carcinoma with transperitoneal approach, Porpiglia and colleagues [15-17] described their experience with direct access to the renal artery while performing transperitoneal radical nephrectomy procedures. However, due to the transperitoneal approach, there are some unavoidable risks with these procedures. For example, the risk of ligation of the superior mesenteric artery would be a fatal mistake for the patient. Retroperitoneoscopy also seems to permit faster access to the renal artery than the transperitoneal approach [15]. In the present study, we attempted fast access and early ligation of the renal vessels. The advantages of fast access and early ligature of renal pedicle can be summarized as follows: reduce the manipulation of renal tumor; reduce the potential risk of malignant cell spread due to reducing manipulation of the kidney before ligating renal vessels; lower the blood loss in further steps of dissection; facilitate the dissection of the kidney in further steps due to less bleeding and loosen the kidney from renal pedicle; and relieve the mental stress of surgeon in the further operation steps.

Owing to the advantages above, our modified technique resulted in less operation time and intraoperative blood loss compared with the previous one. However, there is no doubt that patients in Group 2 were operated on after a certain amount of upper urinary tract laparoscopic urologic experience gained in Group 1 – which we think might have an impact on the results, particularly the operation time. Surgeons’ experience might be suboptimal during operating on patients in Group 1 when compared with Group 2. In addition, a disadvantage of this technique might be difficulty in the presence of hilar and para-aortic metastatic lymph nodes or a large renal mass, which could result in displacement of the renal pedicle’s position.

All procedures were completed and no procedure required conversion to open surgery. Renal vein injury occurred in two patients of Group 1 and in one patient of Group 2 during renal pedicle dissection. Bleeding due to renal vein injury was controlled laparoscopically in all three patients. These observations suggest that the modified technique is safe and feasible for retroperitoneal laparoscopic radical nephrectomy.

## Conclusions

Early ligature using fast access to the renal vessels during retroperitoneal laparoscopic radical nephrectomy contributed to less operation time and intraoperative blood loss compared with the previous technique. In addition, the new technique permits the procedure to be performed following the principles of open radical nephrectomy.

## Competing interests

The authors declare that they have no competing interests.

## Authors’ contributions

YQ and YX conceived the study. CX-S and ZL collected the cases and clinical information. ZZ-H performed the statistical analysis. YQ and DJ performed the literature review and wrote the manuscript. YX supervised the experiments and manuscript writing. All authors read and approved the final manuscript.
